# Predictive accuracy of sFlt-1/PlGF ratio for preeclampsia and adverse outcomes: prospective, multicenter including primary, secondary, and tertiary care institutions, observational study in Japan

**DOI:** 10.1038/s41440-025-02282-0

**Published:** 2025-08-04

**Authors:** Tomomi Yamazaki, Ana Sofia Cerdeira, Yuko Teraoka, Yuriko Oomori, Yurika Mukai, Jun Sugimoto, Iemasa Koh, Kouji Banno, Manu Vatish, Yoshiki Kudo

**Affiliations:** 1 Department of Obstetrics and Gynecology, Graduate School of Biomedical Sciences, Hiroshima University, Hiroshima, Japan; 2 Nuffield Department of Women’s Health and Reproductive Research University of Oxford Level 3, Women’s Center, John Radcliffe Oxford University Hospital Oxford, Oxford, United Kingdom

**Keywords:** Adverse outcomes, Japan, Predictive accuracy, Preeclampsia, The sFlt-1/PlGF

## Abstract

Obstetric care in Japan is organized in 3 levels and half of all deliveries are conducted at primary facilities without neonatal intensive care unit. We evaluated the predictive accuracy of the sFlt-1/PlGF ratio for the onset of preeclampsia and adverse outcomes in pregnant women with suspected preeclampsia at multiple facilities in Japan, including primary facilities. 356 pregnant women from 18 + 0 to 36 + 6 weeks of pregnancy were enrolled. 303 women were included in the final analysis. The negative predictive value for ruling out preeclampsia within 1 week using the cut-off value 38 was 98.4% (95% CI, 96.2–99.3) with a negative likelihood ratio of 0.22 (95% CI, 0.09–0.53). The positive predictive value for ruling in preeclampsia within 4 weeks using the cut-off value 38 was 48.2% (95% CI, 38.0–58.5). The positive predictive value using a cut-off value of 85 was 65.0% (95% CI, 44.1–81.4) with a positive likelihood ratio of 12.21 (95% CI, 5.20–28.80). The positive predictive value for prediction of adverse outcomes within 4 weeks using the cut-off value 38 was 64.8% (95% CI, 53.1–75.0). To clarify the accuracy of the test currently covered by insurance in Japan, we additionally conducted an analysis focusing on pregnant women between 18 + 0 and 35 + 6 weeks, yielding even higher accuracy. Pregnant women with sFlt-1/PlGF ratio >38 should be referred to a higher-level medical institution. The appropriate use of sFlt-1/PlGF supported by a robust collaboration between primary and tertiary care institutions may help to improve perinatal outcomes in Japan.

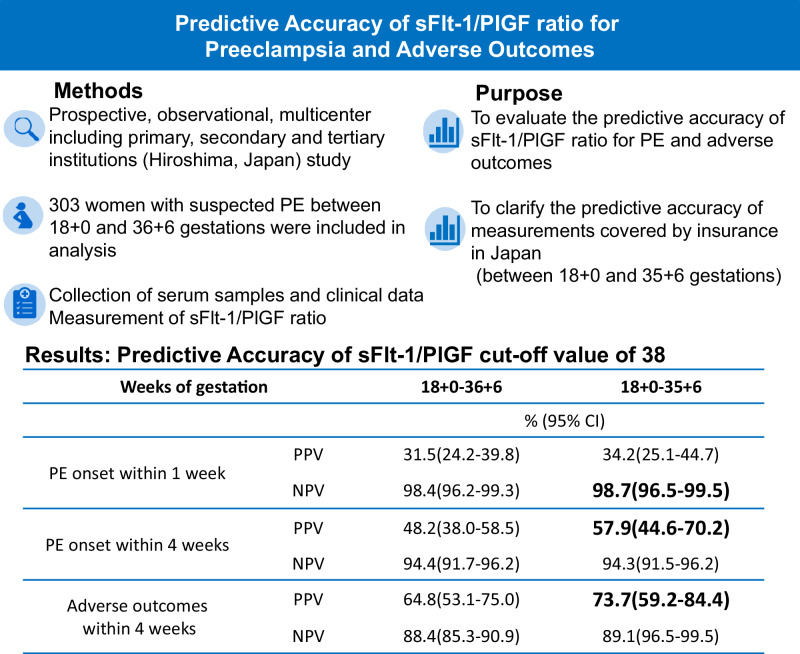

## Introduction

Preeclampsia (PE) is one of the subtypes of hypertensive disorders of pregnancy (HDP). It is a condition characterized by maternal hypertension, accompanied by various clinical symptoms such as proteinuria, organ dysfunction, and uteroplacental dysfunction, and is associated with adverse maternal and fetal outcomes [1–3]. The most common cause of maternal death in Japan is obstetric hemorrhage, followed by neurological disorders closely related to HDP [4, 5]. In addition, the only treatment for PE at present is the delivery of the placenta (i.e. to terminate the pregnancy), and PE is a significant cause of iatrogenic preterm birth [6].

Obstetric care in Japan is organized in 3 levels: primary facilities (medical units for low-risk patients), secondary facilities and tertiary care centers. About half of all deliveries are managed by primary facilities with one to three obstetricians [4]. Primary facilities do not have Neonatal Intensive Care Unit (NICU), and secondary facilities may or may not have NICU depending on the facility. Therefore, primary and secondary facilities refer complex patients to the tertiary centers. Women who were already diagnosed with PE are often transported from primary and secondary facilities to tertiary care centers.

Two prospective multicenter studies (PROGNOSIS and PROGNOSIS Asia) have demonstrated that the sFlt-1 (soluble fms-like tyrosine kinase 1) /PlGF (placental growth factor) ≤38 to rule out PE within 1 week [7, 8]. As a result, the sFlt-1/PlGF ratio test is used as a prospective tool to rule out the development of PE in several countries. In Japan, the measurement of the sFlt-1/PlGF ratio was covered by insurance for high-risk pregnant women at gestation week 18 + 0 to 35 + 6 days since July 2021. The measurement of the sFlt-1/PlGF ratio is a particularly useful test in primary facilities that primarily handle deliveries for low-risk pregnant women. However, most observational studies on the predictive accuracy of the sFlt-1/PlGF ratio conducted in the Japanese population, including PROGNOSIS Asia, have included only patients from tertiary institutions [9, 10]. We therefore considered that a study of pregnant women from primary to tertiary facilities was needed.

In addition to this, currently only the cut-off value of 38 is used as rule out PE within 1 week and rule in PE within 4 weeks in Japan. However, the reported positive predictive value (PPV) for ruling in PE within 4 weeks is not very high (e.g., 36.7% in the validation cohort of PROGNOSIS and 30.3% in PROGNOSIS ASIA) [7, 8], and several studies have reported on the better performance of a cut-off value of 85 for rule in [11–13].

We conducted a prospective, multicenter including primary, secondary, and tertiary care institutions, observational study and evaluated the accuracy of measuring the sFlt-1/PlGF ratio in the Japanese perinatal care system under insurance coverage. We evaluated the currently used cut-off of 38 as well as a cut-off value of 85.

Point of view

- Clinical relevance
  The predictive accuracy for the onset of PE and adverse outcomes using the sFlt-1/PlGF ratio under the current Japanese insurance system was found to be even higher than previously reported. This test may be particularly useful in facilities without NICUs, such as primary care facilities.
- Future direction
  Although the sFlt-1/PlGF ratio is an extremely useful test, it cannot be said that it is currently widely implemented in primary care facilities in Japan. We would like to improve perinatal outcomes through appropriate use of sFlt-1/PlGF and collaboration between primary and tertiary care institutions.
- Consideration for the Asian population
  PROGNOSIS ASIA demonstrated that an sFlt-1/PlGF ratio ≤38 could reliably rule out the onset of PE within 1 week in Asian women. Its role is considered particularly important in regions with limited access to advanced medical facilities such as NICUs.

## Methods

### Study overview

This study was a multicenter prospective, blind, observational study including 3 primary (Kawasaki clinic, Nakagawa clinic, and Yorishima clinic), 4 secondary (Higashihiroshima medical center, Hiroshima red cross hospital, JA Hiroshima general hospital, and Onomichi general hospital) and 1 tertiary care (Hiroshima University Hospital) institutions. None of the primary facilities have NICUs, 2 of the 4 secondary facilities do not have NICUs, 1 facility can handle deliveries from 34 weeks onwards, and 1 facility can handle deliveries from 28 weeks onwards. This study was conducted in collaboration with the University of Oxford. The study was approved by the Ethics Committee for Epidemiological Research of Hiroshima University (No. E-1782). This study was performed in compliance with the guidelines for Good Clinical Practice and applicable local legal and regulatory requirements. We obtained informed consent from each participating patient. Elecsys® sFlt-1 and PlGF reagents used in this study were provided by Roche Diagnostics, Inc. The study protocol was designed by the investigators. Roche Diagnostics, Inc. had no role in data collection, data analysis, or final approval of the manuscript. The authors performed the data analysis and interpretation and wrote the manuscript. All authors were independent of the funder.

### Subjects and procedures

In this study, we included Japanese pregnant women who were 18 years of age or older (18 + 0 to 36 + 6 weeks of gestation at the first visit) with suspected PE between November 2019 and December 2023. The definition of suspected PE includes newly onset hypertension; with systolic blood pressure (SBP) of 130 mmHg or higher and/or diastolic blood pressure (DBP) of 80 mmHg or higher; newly detected proteinuria; with a spot urine protein level of 2+ or a spot urine protein level of 1+ on two or more consecutive occasions; rapid worsening of edema or sudden weight gain; symptoms such as headache, epigastric pain, or visual disturbances suggestive of PE; liver dysfunction or coagulation disorders; and ultrasound findings indicating placental dysfunction, such as fetal growth restriction (FGR) or abnormal umbilical artery blood flow. Pregnant women with existing PE, eclampsia, or HELLP syndrome, as well as those with multiple pregnancies or fetal chromosomal abnormalities were excluded.

We collected maternal serum samples and clinical data over time. Maternal serum levels of sFlt-1 and PlGF were measured using the fully automated Elecsys® sFlt-1 and PlGF immunoassays on the Roche Cobas® 8000 e 801 module at Hiroshima University hospital laboratory after delivery [14].

### Diagnostic criteria

The diagnostic criteria for PE used the definitions and classifications published by JSSHP (Japan Society for the Study of Hypertension Pregnancy) in 2018. PE was defined as new onset of hypertension (BP ≥ 140/90 mmHg) at or after 20 weeks’ gestation and either proteinuria (≥300 mg/24 h or protein/creatinine ratio ≥0.3) or increasing liver transaminase or serum creatinine (in the absence of liver and renal disease respectively), cerebral or visual symptoms, thrombocytopenia, or uteroplacental dysfunction [15].

In previous studies, maternal adverse outcomes were defined as pulmonary edema, acute renal failure, cerebral hemorrhage, and death (other than PE, eclampsia, or HELLP syndrome). Fetal adverse outcomes were defined as perinatal or fetal death, FGR, small for gestational age (SGA), delivery before 34 weeks of gestation, respiratory distress syndrome, intraventricular hemorrhage, necrotizing enterocolitis, and placental abruption [7, 8, 10]. However, as mentioned above, most primary facilities and some secondary facilities in Japan do not have a NICU. Furthermore, we considered that the management of PE, eclampsia and HELLP syndrome in primary facilities is risky and should be avoided. Therefore, in this study we included PE, eclampsia, and HELLP syndrome as maternal adverse outcomes. We also included delivery before 36 weeks of gestation and birth weight under 2200 g, which are the criteria for NICU admission at our hospital, as fetal adverse outcomes.

### Analysis objective

The primary objectives were to evaluate the accuracy of sFlt-1/PlGF ≤38 ruling out PE within 1 week, and the accuracy of sFlt-1/PlGF >38 ruling in PE within 4 weeks, among pregnant women presented with suspected PE in primary, secondary, and tertiary facilities in Japan. Additionally, the accuracy of the cut-off value of 85, which is considered to have a high PPV, was also examined. The secondary objectives were to investigate the accuracy of sFlt-1/PlGF ratio for predicting adverse outcomes within 4 weeks. To clarify the predictive accuracy of testing currently covered by insurance in Japan, we additionally conducted an analysis focusing on pregnant women from 18 + 0 to 35 + 6 pregnancy and an analysis in each group such as primary facilities, secondary facilities, and tertiary center.

### Statistical analysis

Sample size considerations have been based on Pepe et al. [16] and the anticipated performance (as derived from previous studies). The acceptable minimum negative predictive value (NPV) was defined as 90%, and the PPV as 20%. The prevalence of PE in pregnant women suspected of PE was estimated to be ~15% [7, 8, 10]. Based on these assumptions we calculated that a sample size of about 300 patients should be appropriate to provide predictive values with sufficient accuracy. Statistical analysis was conducted using JMP Pro 17.0®. Descriptive statistics were reported as medians and interquartile ranges for continuous data and as absolute and relative frequencies for count data. The predictive performance of the sFlt-1/PlGF ratio for each objective was determined by estimating of NPV, PPV, sensitivity, specificity, negative likelihood ratio, positive likelihood ratio, and area under the receiver-operator characteristic (ROC) curve, each with the corresponding 95% confidence intervals (CI). A *p* value < 0.05 was considered statistically significant. Comparisons of values were performed using the t-test, χ² test, Fisher’s exact test, and Wilcoxon rank-sum test.

## Results

### Study population

Between November 2019 and December 2023, a total of 356 women with suspected PE were enrolled (Supplementary Figure). 53 women were excluded, 40 women had already been diagnosed with PE at the time of enrollment and 13 had multiple-gestation pregnancies. The final analysis included 303 eligible women with 54 enrolled at primary facilities (11 of these were transferred to tertiary center), 108 at secondary facilities (3 of these were transferred to tertiary center), and 141 at a tertiary center. Of the 303 patients analyzed, 21 women (6.9%) were diagnosed with PE within 1 week, and 40 women (13.2%) were diagnosed with PE within 4 weeks after baseline visit. In total, 61 participants were diagnosed with PE and the overall prevalence of PE in this study population was 20.1%. There were no cases of eclampsia. There were no significant differences in age, gestational age, body mass index (BMI) pre-pregnancy and smoking rate at baseline visit between participants who did not develop PE and those who did. The proportion of primiparous women, SBP at baseline visit, and DBP at baseline visit were significantly higher in women who developed PE than in women who did not. The gestational age at delivery was significantly lower in women who developed PE compared to those who did not (Supplementary Table 1). The characteristics of participants in primary facilities, secondary facilities and tertiary center are presented in Table 1. The incidence of PE in each group was 13.0% in primary facilities, 25.0% in secondary facilities, and 19.2% in tertiary center. Participants in secondary facilities and tertiary center were significantly older, had higher BMI, and a higher proportion of comorbidities (e.g., hypertension, diabetes mellitus, gestational diabetes mellitus, SLE, etc.) compared to those in primary facilities.

**Table 1:** Characteristics of participants at primary facilities, secondary facilities and tertiary center

Characteristics	All participants	Primary facilities	Secondary facilities	Tertiary center
	(*N* = 303)	(*n* = 54)	(*n* = 108)	(*n* = 141)
Median age, y (IQR)	34 (29–38)	30 (27–34)	35 (32–37)☨	34 (29–39)†
Primiparous woman (%)	147 (48.5)	30 (55.6)	54 (50.0)	63 (44.7)
Median BMI prepregnancy, kg/m^2^ (IQR)	22.2 (20.1–27.2)	21.2 (19.2–25.8)	22.8 (20.4–27.0)†	22.9 (20.2–28.9)†
Median week of gestation at baseline visit (IQR)	31.9 (26.9–35.0)	31.9 (28.0–35.3)	32.2 (28–34.7)	31.9 (26.3–35.3)
Woman with complications (%)	105 (34.7)	7 (13.0)	40 (37.0)†	58 (41.1)†
Incidence of PE (%)	61 (20.1)	7 (13.0)	27 (25.0)	27 (19.2)
Median week of gestation at delivery (IQR)	37.9 (37.0–39.1)	39.5 (37.4–40.0)	37.7 (37–39)☨	37.6 (36.5–38.9)☨

*BMI* body mass index, *IQR* interquartile range, *PE* preeclampsia

*p* values were calculated with the use of the Wilcoxon rank-sum test for continuous variables and χ2 test for categorical variables

†*p* < 0.05 for the comparison with participants in the primary facilities

☨*p* < 0.001 for the comparison with participants in the primary facilities

### Prediction of PE

**Fig. 1:**
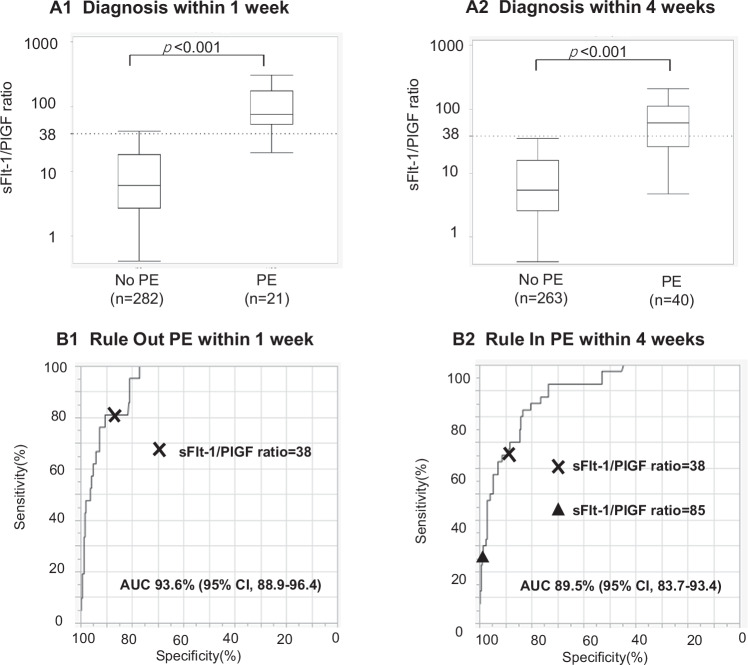
Performance of the sFlt-1/PlGF ratio for prediction of PE between 18 + 0 and 36 + 6 weeks. **A1** Distribution of sFlt-1/PlGF ratio for participants who developed or did not develop PE within 1 week. **A2** Distribution of sFlt-1/PlGF ratio for participants who developed or did not develop PE within 4 weeks; The bottom and top edges of each box represent the first and third quartiles, respectively, the band within the box represents the median value, the whiskers represent values that are 1.5 times the interquartile range, in log-scale. **B1** Performance of the ratio for ruling out PE within 1 week. **B2** Performance of the ratio for ruling in PE within 4 weeks. X shows ratio = 38, and ▲ shows ratio = 85. AUC area under curve, CI confidence interval, PE preeclampsia

The sFlt-1/PlGF was measured in pregnant women with suspected PE from 18 + 0 to 36 + 6 days. The median sFlt-1/PlGF ratio in women who developed PE within 1 week (77.0) was higher compared to women who did not develop PE within 1 week (6.1) (*p* < 0.001). Similarly, the median sFlt-1/PlGF ratio in women who developed PE within 4 weeks (62.0) was higher compared to women who did not develop PE within 4weeks (5.4) (*p* < 0.001) (Fig. 1). The NPV for ruling out PE within 1 week using the cut-off value 38 was 98.4% (95% CI, 96.2–99.3), with a sensitivity of 81.0% (95% CI, 58.1–94.6), a specificity of 86.9% (95% CI, 82.4–90.6) and a negative likelihood ratio of 0.22 (95% CI, 0.09–0.53). The area under curve (AUC) with ROC curve of the sFlt-1/PlGF ratio for ruling out PE within 1 week was 93.6% (95% CI, 88.9–96.4). The PPV for ruling in PE within 4 weeks using the cut-off value 38 was 48.2% (95% CI, 38.0–58.5). The PPV using a cut-off value of 85 was 65.0% (95% CI, 44.1–81.4) with a positive likelihood ratio of 12.21 (95% CI, 5.20–28.80). The AUC of the sFlt-1/PlGF ratio for ruling in PE within 4 weeks was 89.5% (95% CI, 83.7–93.4) (Fig. 1 and Table 2).

**Table 2:** Performance of the sFlt-1/PlGF ratio for prediction of PE in participants with suspected desease between 18 + 0 and 36 + 6 weeks

Cutoff	TN/FN	TP/FP	NPV, %	PPV, %	Sensitivity, %	Specificity, %	Negative LR	Positive LR
			(95% CI)	(95% CI)	(95% CI)	(95% CI)	(95% CI)	(95% CI)
Within 1 week								
38	245/4	17/37	98.4 (96.2–99.3)	31.5 (24.2–39.8)	81.0 (58.1–94.6)	86.9 (82.4–90.6)	0.22 (0.09–0.53)	6.17 (4.28–8.89)
Within 4 weeks								
38	235/14	26/28	94.4 (91.7–96.2)	48.2 (38.0–58.5)	65.0 (48.3–79.4)	89.4 (85.0–92.8)	0.39 (0.26–0.60)	6.11 (4.02–9.27)
85	256/27	13/7	90.5 (88.4–92.2)	65.0 (44.1–81.4)	32.5 (18.6–49.1)	97.3 (94.6–98.9)	0.69 (0.56–0.86)	12.21 (5.20–28.80)

*CI* confidence interval, *FN* false negative, *FP* false positive, *LR* likelihood ratio, *NPV* negative predictive value, *PE* preeclampsia, *PPV* positive predictive value, *TN* true negative, *TP* true positive

### False negative cases

An sFlt-1/PlGF ratio of ≤38 can predict with a very high accuracy (NPV 98.4%), that PE will not develop within 1 week. Among 249 cases with sFlt-1/PlGF ≤38, 4 cases were false negatives in this study (Supplementary Table 2). In all 4 cases, the sFlt-1/PlGF ratio was measured at 35 or 36 weeks of gestations. One patient had obesity (BMI 33.6) and gestational diabetes mellitus, one had type 1 diabetes, and the other two had systemic lupus erythematosus, all of which were accompanied by maternal complications. Due to complications, 3 out of the 4 cases were managed at a tertiary center from early pregnancy. The remaining case was managed at a secondary facility with an NICU. In all cases, there was no severe elevation of blood pressure, and no FGR was observed. Except for the case in which delivery occurred at 35 weeks due to the onset of spontaneous labor, none of the newborns required admission to the NICU.

### Predictive accuracy in line with Japanese insurance coverage

As mentioned above, the sFlt-1/PlGF ratio measurement in Japan is covered by insurance from 18 + 0 to 35 + 6 days of pregnancy. Therefore, the diagnostic accuracy of the test in 267 pregnant women at gestation week 18 + 0 to 35 + 6 days and 204 pregnant women at early gestation week 18 + 0 to 33 + 6 is also shown as a sub analysis (Table 3).

**Table 3:** Performance of the sFlt-1/PlGF ratio using the cut-off value 38 for prediction of PE

Gestational phase	TN/FN	TP/FP	NPV, %	PPV, %	Sensitivity, %	Specificity, %	Negative LR	Positive LR
			(95% CI)	(95% CI)	(95% CI)	(95% CI)	(95% CI)	(95% CI)
Within 1 week								
18 + 0–36 + 6	245/4	17/37	98.4 (96.2–99.3)	31.5 (24.2–39.8)	81.0 (58.1–94.6)	86.6 (82.1–90.4)	0.22 (0.09–0.53)	6.17 (4.28–8.89)
**18** + **0–35** + **6**	**226/3**	**13/25**	**98.7** **(96.5–99.5)**	**34.2** **(25.1–44.7)**	**81.3** **(54.4–96.0)**	**90.0** **(85.7–93.5)**	**0.21** **(0.08–0.58)**	**8.16** **(5.25–12.67)**
18 + 0–33 + 6	182/0	6/16	100.0 (98.0–100.0)	27.3 (19.0–37.5)	100.0 (98.0–100.0)	91.9 (87.2–95.3)	0.00	12.38 (7.74–19.80)
Within 4 weeks								
18 + 0–36 + 6	235/14	26/28	94.4 (91.7–96.2)	48.2 (38.0–58.5)	65.0 (48.3–79.4)	89.4 (85.0–92.8)	0.39 (0.26–0.60)	6.11 (4.02–9.27)
**18** + **0–35** + **6**	**216/13**	**22/16**	**94.3** **(91.5–96.2)**	**57.9** **(44.6–70.2)**	**62.9** **(44.9–78.5)**	**93.1** **(89.0–96.0)**	**0.40** **(0.26–0.61)**	**9.11** **(5.33–15.59)**
18 + 0–33 + 6	176/6	12/10	96.7 (41.0–86.7)	54.6 (37.7–70.4)	66.7 (41.0–86.7)	94.6 (90.3–97.4)	0.35 (0.18–0.68)	12.40 (6.25–24.62)

The gestational age for which the measurement of the sFlt-1/PlGF ratio is covered by insurance in Japan are highlighted in bold

*CI* confidence interval, *FN* false negative, *FP* false positive, *LR* likelihood ratio, *NPV* negative predictive value, *PPV* positive predictive value, *PE* preeclampsia, *TN* true negative, *TP* true positive

When the sFlt-1/PlGF was measured in pregnant women from18 + 0 to 35 + 6 days, the NPV for ruling out PE within 1 week using the cut-off value 38 was 98.7% (95% CI, 96.5–99.5), with a sensitivity of 81.3% (95% CI, 54.4–96.0) and a specificity of 90.0% (95% CI, 85.7–93.5). The NPV for ruling out PE within 4 weeks using the cut-off value 38 was 94.3% (95% CI, 91.5–96.2). The PPV for ruling in PE within 4 weeks using the cut-off value 38 was 57.9% (95% CI, 44.6–70.2).

### Prediction of adverse outcomes

When the sFlt-1/PlGF was measured in pregnant women from18 + 0 to 36 + 6 days, the number of pregnant women with adverse outcomes, combining maternal adverse outcomes (including PE, HELLP syndrome, eclampsia, as well as pulmonary edema, acute renal failure, cerebral hemorrhage, and death) and fetal adverse outcomes (perinatal or fetal death, FGR, SGA, delivery before 36 weeks of gestation, birth weight under 2200 g, respiratory distress syndrome, intraventricular hemorrhage, necrotizing enterocolitis, and placental abruption) within 4 weeks, was 64. The PPV for adverse outcomes within 4 weeks using the cut-off value 38 was 64.8% (95% CI, 53.1–75.0). The PPV using a cut-off value of 85 was 75.0% (95% CI, 53.1–88.8). When the sFlt-1/PlGF was measured in pregnant women from 18 + 0 to 35 + 6, the PPV for adverse outcomes within 4 weeks using the cut-off value 38 was 73.7% (95% CI, 59.2–84.4). The PPV using a cut-off value of 85 was 77.8% (95% CI, 54.6–91.1) (Table 4).

**Table 4:** Performance of the sFlt-1/PlGF ratio for prediction of adverse outcomes (including PE and NICU admission) within 4 weeks

Gestational phase	TN/FN	TP/FP	NPV, %	PPV, %	Sensitivity, %	Specificity, %	Negative LR	Positive LR
			(95% CI)	(95% CI)	(95% CI)	(95% CI)	(95% CI)	(95% CI)
**Cut-off 38**								
18 + 0–36 + 6	220/29	35/19	88.4 (85.3–90.9)	64.8 (53.1–75.0)	54.7 (41.8–67.2)	92.1 (87.9–95.2)	0.49 (0.38–0.65)	6.88 (4.23–11.18)
**18** + **0–35** + **6**	**204/25**	**28/10**	**89.1** **(96.5–99.5)**	**73.7** **(59.2–84.4)**	**52.8** **(38.6–66.7)**	**95.3** **(91.6–97.7)**	**0.49** **(0.37–0.66)**	**11.31** **(5.86–21.80)**
18 + 0–33 + 6	171/11	16/6	94.0 (90.8–96.1)	72.7 (53.4–86.1)	59.3 (38.8–77.6)	96.6 (92.8–98.8)	0.42 (0.27–0.67)	17.48 (7.50–40.80)
**Cut-off 85**								
18 + 0–36 + 6	234/49	15/5	82.7 (80.6–84.6)	75.0 (53.1–88.8)	23.4 (13.8–35.7)	97.9 (95.2–99.3)	0.78 (0.68–0.90)	11.20 (4.23–29.67)
18 + 0–35 + 6	210/39	14/4	98.1 (95.3–99.5)	77.8 (54.6–91.1)	26.4 (15.3–40.3)	98.1 (95.3–99.5)	0.75 (0.64–0.88)	14.13 (4.85–41.19)
18 + 0–33 + 6	174/17	10/3	91.1 (88.5–93.2)	76.9 (49.5–91.9)	37.0 (19.4–57.6)	98.3 (95.1–99.7)	0.64 (0.48–0.86)	21.85 (6.42–74.39)

The gestational age for which the measurement of the sFlt-1/PlGF ratio is covered by insurance in Japan are highlighted in bold

*CI* confidence interval, *FN* false negative, *FP* false positive, *LR* likelihood ratio, *NPV* negative predictive value, *PPV* positive predictive value, *TN* true negative, *TP* true positive

Furthermore, when the sFlt-1/PlGF was measured in pregnant women from 18 + 0 to 35 + 6, performance for prediction of adverse outcomes within 4 weeks in each group such as primary facilities, secondary facilities, and tertiary center is shown (Table 5). In primary facilities, the PPV for adverse outcomes within 4 weeks using the cut-off value 38 was 83.3% (95% CI, 40.2–97.4). The PPV using a cut-off value of 85 was 100.0% (95% CI, 15.8–100.0). There were 6 cases with sFlt-1/PlGF >38 at primary facilities (Supplementary Table 3). The sFlt-1/PlGF ratio was not determined before delivery, but all cases were transferred to a tertiary center during pregnancy. There were 17 cases with sFlt-1/PlGF >38 at secondary facilities. Among these, one case where the sFlt-1/PlGF ratio was measured at 26 weeks due to FGR (sFlt-1/PlGF: 1032), and another case where the ratio was measured at 30 weeks due to elevated blood pressure (sFlt-1/PlGF: 356), later developed early-onset PE and were transferred to tertiary center.

**Table 5:** Performance of the sFlt-1/PlGF ratio for prediction of adverse outcomes (including PE and NICU admission) within 4 weeks of primary facilities, secondary facilities and tertialy center between 18 + 0 and 35 + 6 weeks

Facilities	TN/FN	TP/FP	NPV, %	PPV, %	Sensitivity, %	Specificity, %	Negative LR	Positive LR
			(95% CI)	(95% CI)	(95% CI)	(95% CI)	(95% CI)	(95% CI)
**Cut-off 38**								
primary facilities	38/3	5/1	92.7 (83.8–96.9)	83.3 (40.2–97.4)	62.5 (24.5–91.5)	97.4 (86.5–99.9)	0.38 (0.16–0.94)	24.37 (3.27–181.52)
secondary facilities	72/9	11/6	88.9 (83.1–92.9)	64.7 (43.6–81.3)	55.0 (31.5–77.0)	92.3 (84.0–97.1)	0.49 (0.30–0.79)	7.15 (3.01–16.98)
terciary center	94/13	12/3	87.9 (83.2–91.4)	80.0 (55.0–92.9)	48.0 (27.8–68.7)	96.9 (91.2–99.4)	0.54 (0.37–0.78)	15.52 (4.74–50.83)
**Cut-off 85**								
primary facilities	39/6	2/0	86.7 (81.3–84.6)	100.0 (15.8–100.0)	25.0 (3.2–65.1)	100.0 (91.0–100.0)	0.75 (0.50–1.12)	–
secondary facilities	74/16	4/4	82.2 (78.7–85.3)	50.0 (21.5–78.6)	20.0 (5.7–43.7)	94.9 (87.4–98.6)	0.84 (0.67–1.06)	3.90 (1.07–14.25)
terciary center	97/17	8/0	85.1 (81.4–88.2)	100.0 (63.1–100.0)	32.0 (15.0–53.5)	100.0 (96.3–100.0)	0.68 (0.52–0.89)	–

*CI* confidence interval, *FN* false negative, *FP* false positive, *LR* likelihood ratio, *NPV* negative predictive value, *PPV* positive predictive value, *TN* true negative, *TP* true positive

## Discussion

In this study, we evaluated the predictive accuracy of the sFlt-1/PlGF ratio using cut-off values of 38 and 85 for the onset of PE and adverse outcomes in pregnant women with suspected PE at multiple facilities in Japan, including primary facilities.

Consistent with previous reports, the sFlt-1/PlGF ratio cut-off of value 38 had a high NPV for ruling out PE within 1 week in high-risk pregnant women with a low negative likelihood ratio. Additionally, the NPV for ruling out PE within 4 weeks was also high. Currently, the measurement of sFlt-1/PlGF is covered by insurance for pregnant women between 18 + 0 and 35 + 6 of gestation in Japan. The currently reported NPV to date is based on studies involving pregnant women up to 36 + 6 days. Our secondary analysis on pregnant women up to 35 + 6 days, (i.e. covered by insurance) showed that the NPV remained very high in this population. The PPV is influenced by the prevalence of the disease. In this study, we report a higher PPV of the sFlt-1/PlGF ratio compared to previous reports. This may be attributed to the higher incidence rate of PE among participants (20.1%) relative to earlier studies (e.g., 17.8% in the validation cohort of PROGNOSIS and 14.4% in PROGNOSIS ASIA) [7, 8].

It has been previously reported that an elevated sFlt-1/PlGF ratio is more pronounced in early-onset PE [17, 18]. In this study as well, all false negative pregnant women with sFlt-1/PlGF ≤38 who developed preeclampsia within 1 week had their measurements taken at or beyond 35 weeks of gestation. In addition, all 4 cases that resulted in false negatives involved mothers with complications such as obesity, gestational diabetes mellitus, type 1 diabetes, or systemic lupus erythematosus, suggesting the presence of pre-existing maternal vascular endothelial dysfunction. All cases were managed at facilities with NICUs due to complications. Although all cases were diagnosed with PE within 1 week, none showed a severe elevation of blood pressure, and no FGR was observed. Except for the case in which delivery occurred at 35 weeks due to the onset of spontaneous labor, none of the newborns required admission to the NICU.

Currently the cut-off value of 38 is used to rule out PE within 1 week and is used to rule in PE within 4 weeks. However, the reported PPV for ruling in PE within 4 weeks is not very high (e.g., 36.7% in the validation cohort of PROGNOSIS and 30.3% in PROGNOSIS ASIA) [7, 8], and several studies on the cut-off value of 85 have been reported [11–13]. In fact, in this study, the PPV for ruling in PE within 4 weeks using the cut-off value 85 was 65.0% (95% CI, 44.1–81.4), higher than 48.2% (95% CI, 38.0–58.5) using the cut-off value 38 for high-risk pregnant women between 18 + 0 and 36 + 6 of gestation. We therefore would like to suggest the introduction of the cut-off of 85 to assist with ruling in the development of the disease.

On the other hand, in Japan, many deliveries take place at facilities that do not have a NICU. When a new adverse outcome was defined to include PE and NICU admission due to gestational age under 36 weeks or birth weight under 2200 g, the PPV for adverse outcomes within 4 weeks using the cut-off value 38, measured within the insurance-covered range up to 35 + 6 days of gestation, was 73.7% (95% CI, 59.2–84.4). In other words, 3 out of 4 pregnant women required delivery at an advanced medical facility, including NICU admission. When limited to primary facilities that primarily manage low-risk pregnant women, the PPV for adverse outcomes within 4 weeks using the cut-off value 38 was even higher, at 83.3% (95% CI, 40.2–97.4). Considering these results, as is already recommended at present pregnant women with risk factors for PE and an sFlt-1/PlGF ratio >38 should be referred to advanced medical facilities [19]. This is particularly important for facilities without a NICU.

In this study, sFlt-1/PlGF ratio was measured after delivery and was not used for decision-making. However, considering that this study, which included primary to tertiary care facilities, showed such high NPV and PPV, it is sufficiently feasible to use the ratio for decision-making. In particular, it has been shown that measurement under Japan’s insurance coverage and measurement at primary facilities without a NICU were shown to be very effective, with even higher accuracy than the previously reported NPV and PPV of adverse outcomes.

The measurement of the sFlt-1/PlGF ratio is a particularly useful test in primary care facilities that primarily handle deliveries for low-risk pregnant women. However, currently in Japan, measuring the sFlt-1/PlGF ratio for high-risk pregnancies is not yet widely implemented, especially in primary care facilities. In this study, there were 40 pregnant women who had already been diagnosed with PE and transferred at baseline visit or were diagnosed with PE on the same as the transfer; these cases were excluded from the analysis (Supplementary Figure). Among these 40 women, 23 women delivered on the same day or the following day. Furthermore, even pregnant women diagnosed with early-onset PE, 10 out of 21 women required delivery on the same day or the next day, leaving some cases where steroid administration could not be completed in time. Among these, there were pregnant women who had been previously diagnosed with FGR.

PE is a disease that develops and worsens rapidly. In Japan, where half of all deliveries are conducted at primary facilities without NICUs, it is crucial that physicians at these facilities also fully recognize the risks. Pregnant women exhibiting signs of PE, such as hypertension, FGR, or maternal organ dysfunction, should be tested for sFlt-1/PlGF. Pregnant women with sFlt-1/PlGF ratio >38, especially those with cases before 34 weeks of gestation, should be promptly referred to a tertiary center. Based on the results of this study, we would like to raise awareness among physicians at primary care facilities about the importance of predicting the onset of PE. We would like to improve perinatal outcomes in Japan through appropriate use of sFlt-1/PlGF and collaboration between primary and tertiary care institutions.

### Asian perspectives

Following the PROGNOSIS study [7], the PROGNOSIS ASIA study [8] demonstrated that an sFlt-1/PlGF ratio ≤38 could reliably rule out the onset of PE within 1 week in Asian women. As a result, the sFlt-1/PlGF ratio is increasingly recognized across clinical settings in Asia as a biomarker for predicting and managing PE [19–22].

The use of the sFlt-1/PlGF test varies significantly by region due to differences in medical infrastructure and economic conditions. However, its role is considered particularly important in regions with limited access to advanced medical facilities such as NICUs.

## Conclusion

This study evaluated the predictive accuracy of sFlt-1/PlGF ratio for preeclampsia and adverse outcomes in multicenter including primary, secondary, and tertiary institutions in Japan. The predictive accuracy in Japanese perinatal care system under insurance coverage was found to be even higher than previously reported.

## Supplementary information

Supplementary table 1

Supplementary table 2

Supplementary table 3

Supplementary Figure
