# Supplementary material for: Predictive accuracy of sFlt-1/PlGF ratio for preeclampsia and adverse outcomes: prospective, multicenter including primary, secondary, and tertiary care institutions, observational study in Japan
Source: Hypertens Res. 2025 Aug 4;48(10):2548–57. doi: 10.1038/s41440-025-02282-0 (PMC12497639; doi:10.1038/s41440-025-02282-0)
Supplement: Supplementary file 4 — Supplementary Figure [file 41440_2025_2282_MOESM4_ESM.pptx]

## Slide 1
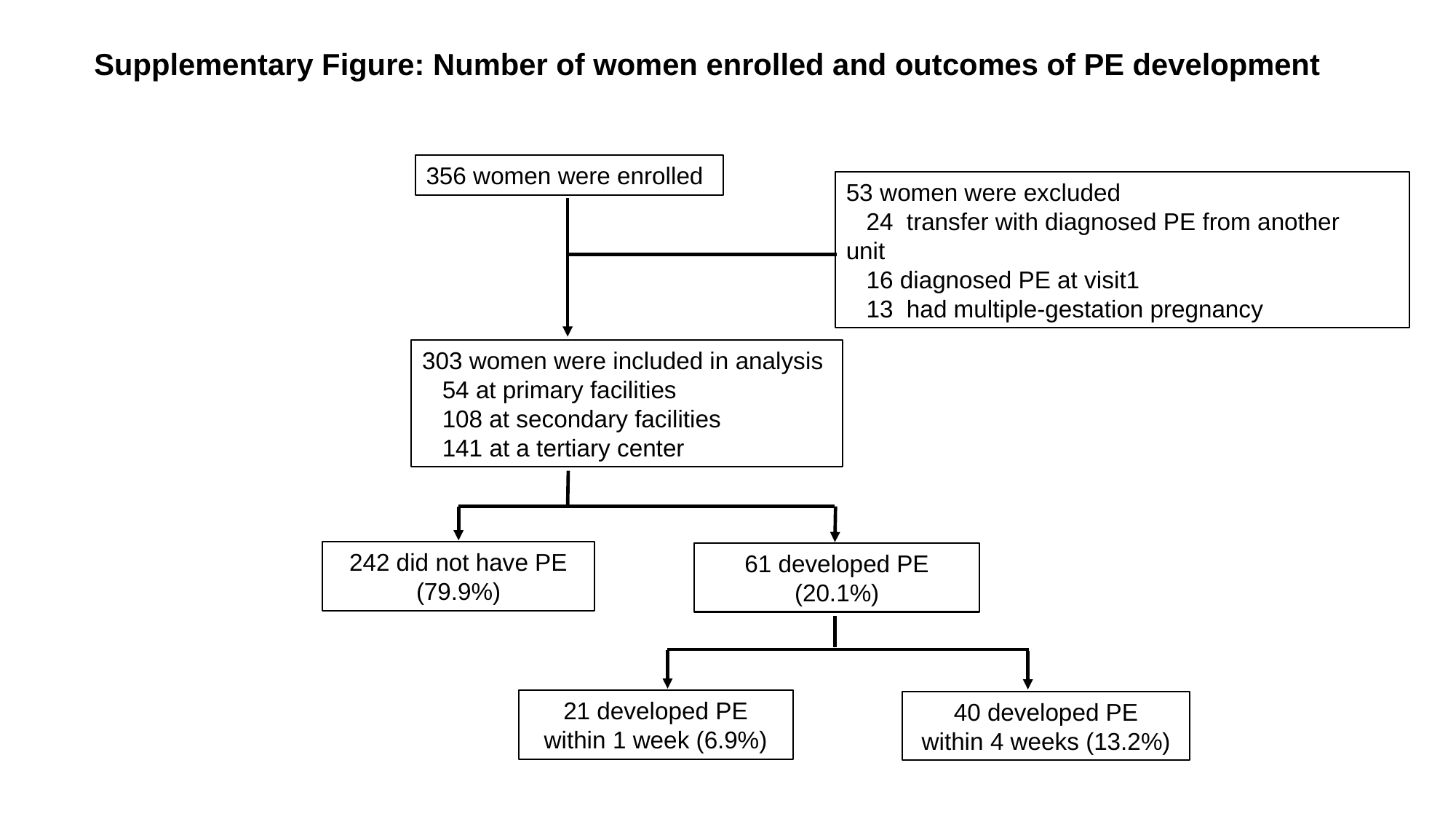

Supplementary Figure: Number of women enrolled and outcomes of PE development
356 women were enrolled
53 women were excluded
 24 transfer with diagnosed PE from another unit
 16 diagnosed PE at visit1
 13 had multiple-gestation pregnancy
303 women were included in analysis
 54 at primary facilities
 108 at secondary facilities
 141 at a tertiary center
242 did not have PE
(79.9%)
61 developed PE
(20.1%)
21 developed PE
within 1 week (6.9%)
40 developed PE
within 4 weeks (13.2%)
